# MOTIVATING OLDER ADULTS THROUGH IMMERSIVE VIRTUAL EXERCISE (MOTIVE)

**DOI:** 10.1093/geroni/igae098.1903

**Published:** 2024-12-31

**Authors:** Brittany Burch, Barbara Resnick, Jimmy Reyes

**Affiliations:** University of Maryland Baltimore, Baltimore, Maryland, United States; University of Maryland Baltimore, Baltimore, Maryland, United States; University of Northern Iowa, Cedar Falls, Iowa, United States

## Abstract

Physical activity among older adults can prevent and treat a host of chronic conditions and reduce mortality. However, people who are high-income are almost twice as likely to meet the physical activity guidelines compared to their low-income peers. Physical activity interventions are a critical approach to directly decrease health disparities among older adults who are low-income. We present pilot study results from an innovative intervention titled Motivating Older Adults Through Immersive Virtual Exercise (MOTIVE), which aimed to: 1) determine the feasibility of MOTIVE and 2) test the preliminary efficacy of MOTIVE at increasing physical activity among older adults who are low-income and living in urban environments. We randomized 10 older adults in Baltimore to the fully-immersive virtual reality exercise intervention (MOTIVE) and 10 to a physical activity education only control group. For 8 weeks, twice weekly, the participants in the MOTIVE intervention group biked through virtual naturalistic environments and smashed targets to the beat of their favorite music. To analyze the data, we performed descriptive statistics and used linear mixed models, controlling for covariates. We found that MOTIVE group participants attended an average of 15 out of the 16 sessions. During the 8 weeks, according to the Yale Physical Activity Survey Part 2, the MOTIVE participants significantly increased their physical activity (p<.001, CI=-31.3,-11.3). While the interaction of time and group was not statistically significant, the Cohen’s d (.53) indicated a moderate effect, favoring the MOTIVE intervention group. These results provide strong preliminary data to support a scaled-up study.

